# Maxillofacial Bone Fractures in Children and Adolescents in Saudi Arabia: A Systematic Review

**DOI:** 10.7759/cureus.60765

**Published:** 2024-05-21

**Authors:** Rahul Gaikwad, Mishal Almutairi, Anas Al-Moshiqah, Fida Almutairi, Abdullah Alharbi, Abdullah Alhudaithi, Abdullah A Alayouni, Abdulrahman M Alharbi, Sultan Algefari

**Affiliations:** 1 Community Dentistry and Oral Epidemiology, College of Dentistry, Qassim University, Buraydah, SAU; 2 College of Dentistry, Qassim University, Buraydah, SAU; 3 Dentistry, Qassim University, Buraydah, SAU

**Keywords:** trauma, dentistry, child, young adults, epidemiology

## Abstract

Facial injuries, including maxillofacial trauma (MFT), are common in children and adolescents due to their age and bone maturation stage. Children's injuries are less common than adults' due to parental supervision and the flexibility of the facial bone. Causes of maxillofacial bone fractures (MFBF) vary based on socioeconomic, cultural, and environmental factors. Management of MFBF in children and adolescents should consider their growth and development stage. A systematic review is needed to understand the prevalence, pattern, and distribution of MFBF in Saudi Arabia.

This systematic review aimed to identify papers on MFBF in children and adolescents in Saudi Arabia using the Preferred Reporting Items for Systematic reviews and Meta-Analyses (PRISMA) statement. The search strategy involved searching electronic databases like PubMed/Medline, Web of Science, and Ebsco. The review included full-text original research papers, with inclusion criteria including English publications, human studies, and no restrictions on sample size, gender, date, or language. The selection process involved screening titles and abstracts, evaluating full texts, and identifying relevant studies. Data extraction involved two authors individually assessing selected studies.

The PRISMA flow diagram of the literature search revealed that 26 papers were identified, of which 15 remained after excluding duplicates. After screening titles and abstracts, 10 articles were removed, and five papers were assessed for eligibility. Four papers met the inclusion criteria for the systematic review. The studies examined 1447 patients for the presence of MFBF in different regions. The majority of MFBF were caused by falls and road traffic accidents (RTAs) in children and adolescents. Mandibular fractures were the most common, followed by maxillary fractures. The majority of patients had tooth/teeth avulsions, followed by tooth luxation and crown fractures. Only one study described investigation methods for MFBF diagnosis.

The systematic review reveals a high prevalence of MFBF among children and adolescents in Saudi Arabia, primarily due to falls and RTAs. The mandible is the most frequently fractured bone, and many children have concomitant teeth involvement. To reduce MFBF, effective initiatives and parental awareness strategies are recommended.

## Introduction and background

Facial injuries are described as any injury to the face, including damage to soft tissues, bones, blood vessels, nerves, and other facial structures. Maxillofacial trauma (MFT) follows trauma to the face, head, and jaws, and 51% of road traffic accidents (RTAs) lead to MFT [1]. These fractures may cause blood loss and airway obstruction and can be fatal in some cases [2]. Previous studies also show long-term psychological impacts of MFT [3, 4].

Children are unique due to their age and the level of bone development. In relation to injury, their characteristics of oral and maxillofacial bone fractures (MFBF) are different [5], but injury is much less common in children than in adults due to parental supervision [6]. The reduced incidence of facial fractures in children compared to adults is probably due to the flexibility of the facial bones, lack of pneumatization of the paranasal sinuses, and protection of the malar region by the prominent buccal pad of fat in infants [7]. The etiology and patterns of children and adolescents' MFBF differ from adults due to anatomical variables, growth, and variations in socioeconomic, cultural, and environmental factors. Maxillofacial injuries in children and teenagers are common and can result from low-velocity incidents like falls, sports, or violence, as well as high-velocity incidents like RTAs or pellet injuries. Maxillofacial injuries in children are commonly linked to their excessive physical activity, lack of supervision, propensity for risk-taking behavior, and other variables.

Malocclusion in children can be exacerbated by an interruption in typical dental growth, particularly throughout the primary and mixed dentition stages. However, a spontaneous correction of occlusal malalignment may occur in children as the deciduous teeth are shed and replaced by permanent teeth [8]. Causes of fractures are closely linked with age-related levels of activity and vary depending on the data source [9].

Management of children and adolescents' MFBF should be tailored to account for their growth and development stages. Understanding the features of MFBF in children and adolescents can assist in making precise diagnoses and selecting suitable treatment approaches. Currently, there is a lack of information on maxillofacial injuries in pediatric and adolescent patients in Saudi Arabia. Research in many provinces is limited [10, 11, 12]; however, no systematic review has been available in the literature to date. Therefore, to gain a clearer picture from the available evidence, the present systematic review aimed to examine the prevalence, pattern, and distribution of MFBF in children and adolescent patients in Saudi Arabia.

## Review

The current systematic review was conducted using the principles outlined in the Preferred Reporting Items for Systematic Reviews and Meta-Analyses (PRISMA) statement [13]. A search strategy was created to find papers on maxillofacial bone fractures in children and adolescents in Saudi Arabia. The following key terms and combinations were used to conduct a database search: ((((ALL=(Maxillofacial Fracture)) OR ALL=(Maxillofacial Trauma)) AND ALL=(children)) AND ALL=(adolescents)) AND ALL=(Saudi Arabia)). Electronic databases such as PubMed/Medline, Web of Science, and Ebsco were searched on March 20, 2024. All pertinent papers were carefully reviewed in the reference list to find any overlooked articles.

Inclusion and exclusion criteria

All full-text original research papers that provided information on MFBF in children and adolescents in Saudi Arabia were included. The initial selection criteria included articles in English with no time restrictions and experiments conducted in humans. There were no limitations on sample size, gender of participants, publication date, or language. Excluded were review articles, case reports, commentaries, letters to the editor, novels, and unpublished articles.

Study selection

Two authors conducted an initial screening of titles and abstracts to identify relevant papers. Every potentially qualified manuscript was thoroughly examined and assessed to determine which research met all the inclusion requirements. Any disagreements were settled by discussion with a third author, and a final list of the research papers to be included in this evaluation was confirmed.

Data extraction

Two authors independently evaluated all chosen papers to collect data, including author information, journal, patient demographics (age and gender), etiology, MFBF pattern, and treatment methods. All uncertainties were addressed and resolved during consensus discussions involving all authors.

Results

**Figure 1:**
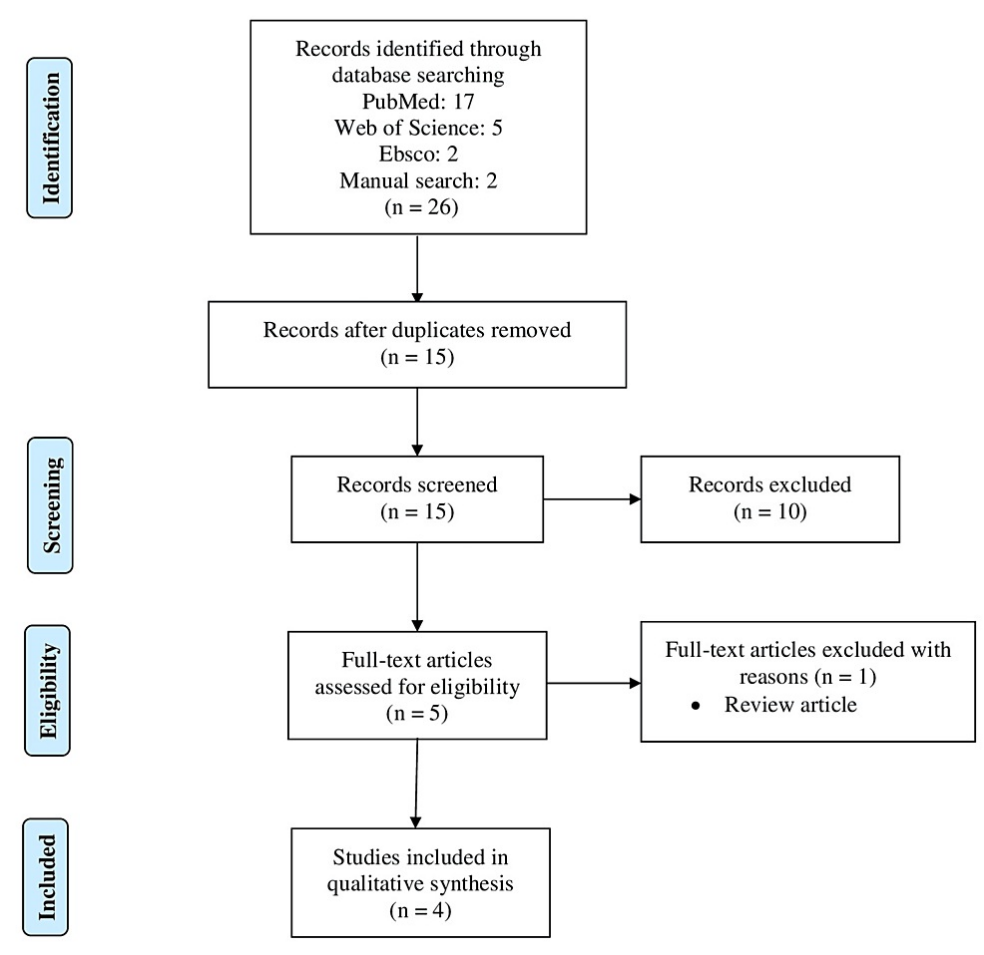
Preferred Reporting Items for Systematic reviews and Meta-Analyses.

Figure 1 displays the PRISMA flow diagram illustrating the literature search process and outcomes. We found 26 papers by an initial search across various electronic databases (PubMed: 17, Web of Science: 5, Ebsco: 2) and 2 papers through manual hand-searching. After removing duplicates, 15 papers remained. Ten publications were excluded after evaluating their titles and abstracts for relevancy. Two reviewers (SG and AG) thoroughly evaluated the full-text articles of the remaining 5 papers to determine their eligibility, resulting in the exclusion of one article. Finally, we included four papers [10-12, 14] that fulfilled the inclusion criteria for this present systematic review.

Study characteristics

The detailed characteristics of all included studies are given in Table 1. All the studies [10-12, 14] were original papers - three retrospective studies and one prospective study - published between 2009 and 2021. Of the included studies, each was conducted in a different region, including one each in Najran [10], Riyadh [11], Madinah [12], and Jeddah [14]. In total, 1447 patients were examined in all four studies for the presence of MFBF [10-12, 14]. Among the 1447 patients examined, MFBF was found in a total of 356 patients, with the number of patients per study ranging from 11 to 247, estimating a prevalence of 24.6%. Across the included studies, two studies [10, 12] reported the gender of MFBF patients, demonstrating a male predilection. The majority of the MFBF cases were caused by falls and RTAs in children and adolescents.

Table 1 illustrates the distribution of fractured maxillofacial bones. Maxillofacial fractures were most frequently found in the mandible, followed by the maxilla. Other common bones involved were zygoma, frontal, orbit, nasal and palate [10-12, 14]. Involvement of the teeth in the maxillofacial injury was reported by three studies [11,12,14], among whom majority of patients had tooth/teeth avulsions, followed by tooth luxation and crown fractures. Only a single study by Shehri SZ et al. [11] described investigation methods for diagnosis of MFBF that included plan radiography, CT scan and CT with contrast. All the included studies except Albeshir H et al. [12] reported definitive treatment modalities as open reduction and internal fixation (ORIF), splinting with arch bar and fixation.

**Table 1:** Characteristics of all included studies. MFBF: Maxillofacial bone fracture; CS: Cross-sectional study; RS: Retrospective study; RTA: Road traffic accidents; PS: Prospective study; ORIF: Open reduction and internal fixation.

Sr no.	Author	Journal/Year	Location	Study type	Sample size	No. of patients with MFBF	Gender	Common causes of injury	Common MFB affected	Teeth involvement	Main treatment modalities
1.	Daniels JS et al. [10]	Craniomaxillofac trauma and Reconst/2021	Najran	RS	247	247	M- 233, F-14	Falls, RTA	Mandible-61.1%, Maxilla-17%, Zygoma-14.6% Orbit-6.1% Frontal-0.4% Palate-0.1%	NR	ORIF
2.	Shehri SZ et al. [11]	Saudi Dent J/2021	Riyadh	RS	223	11	NR	Falls, RTA	Mandible-11 patients	57	Splinting with arch bar
3.	Albeshir H et al. [12]	J Public Health Policy Plann/2018	Madinah	RS	865	85	M- 61, F-24	RTA, Falls	Mandible -70.58%, Maxilla-4.70%, Zygoma-7.05% Orbit-11.76% Frontal-3.52% nasal-2.35%	62	NR
4.	Al-Malik M [14]	J Maxillofac Oral Surg/2009	Jeddah	PS	112	13	NR	Falls, collisions	Mandible -6 patients, Maxilla- 2 patients, Zygoma-1 patient, Orbit-2 patients, Buccal plate-1 patient, combined bones-1 patient	80	fixation

Discussion

The WHO defines adolescence as the time of individual growth and development that occurs prior to adulthood but after childhood, usually between the ages of 10 and 19. Pediatric patients are considered to be from birth until the age of 18 [15]. Diagnosis and treatment planning are crucial steps in treating patients, especially pediatric patients, due to their growth and development. Thus, they require unique attention in order to optimize treatment [16]. The causes and occurrence of MFBF in children and adolescents fluctuate according to their age and gender. The managing oral and maxillofacial surgeon must understand the potential impact of injury and therapy on the growing maxillofacial skeleton.

In the current study, we set out to find the prevalence, pattern, and distribution of MFBF in children and adolescent patients in Saudi Arabia. The results obtained showed a high prevalence (24.6%) of MFBF in a representative sample of children and adolescents included in the studies that make up this systematic review. The majority of the MFBF cases were noticed in males, in the mandible, followed by the maxilla, and were caused by falls and RTAs in children and adolescents.

The incidence of MFBF in children and adolescents has been frequently specified in the literature [17]. Numerous modifications and judgments have been provided on the genesis, occurrence, and therapy of this unique group of patients [18]. In a number of studies, the prevalence of MFBF in the pediatric population ranges from 1.0% to 15.0% in children under the age of 16 [19, 20], and 0.87% to 1.0% in children under the age of 5 [21]. The diminished occurrence among children under the age of five has been related to their decreased physical activity level, less weight, and greater parental supervision, leading them to fall less frequently [22]. The new study has shown a much higher prevalence rate of 24.6% compared to earlier research. The elevated rate in a specific group in Saudi Arabia could be due to the increased participation of male children in outdoor activities including contact sports, cycling, and early exposure to driving at a young age.

This review has illustrated a higher incidence of MFBF in males compared to females. Worldwide, the rate of maxillofacial bone fractures occurs more frequently in males than in females [23, 24]. Almasri [25], in a study conducted in the Southern Aseer region of Saudi Arabia, revealed a male-to-female ratio of 10:1 and found it to be quite high when compared with prior studies from other regions of the Kingdom, primarily Jeddah (4:1) [26] and Al-Madina (4.8:1) [12]. The reason behind this might be because some regions of Saudi Arabia, especially the southern regions, exhibit a highly conservative and cultural humanity, so females utilize a greater amount of time indoors with very little outdoor activity, in contrast to males who spend greater time in cars for transportation and pleasure [25]. In addition, due to the earlier restriction on women driving in Saudi Arabia, they were less likely to be involved in accidents. With the new authorization for women to drive in the country, the ratio may soon change.

The current review observed falls and RTAs as the main causes of MFBF in children and adolescents. Falls have been reported as the etiology for children below five years [10]. RTAs have been demonstrated to increase with the age of the patients. Caregivers are encouraged to keep their children in the back seats of cars and trucks, properly secured with seat belts, child seats, and motorbike or biking protective headgear [27]. The majority of citizens and residents in the kingdom have access to cars, either rented or privately owned, especially the young [28]. Reckless driving, non-compliance with traffic regulations, and driving by unlicensed teens can further increase the occurrence of road traffic accidents.

The mandible has been observed to be the most commonly fractured maxillofacial bone in this review. Past literature in other parts of the world also demonstrated similar findings [7, 21]. Other facial bones involved were the maxilla, zygoma, orbit, and nasal bones. Midface fractures in children and adolescents are rare with high-impact velocity forces because most of the impact is received by the skull due to the small face and big cranium [7]. Daniels JS et al. documented that more midface involvement with fractures occurs in adults as this ratio reverses with increasing age [10]. Three studies included in the present review demonstrated teeth trauma in the form of tooth/teeth avulsions followed by tooth luxation and crown fractures in patients with MFBF. Past studies also noted similar findings [29]. Díaz JA et al. exhibited that subluxation followed by avulsion is more common in the primary dentition [29], and others reported maxillary incisors as the commonly fractured teeth among pediatric patients [30].

Strengths of the review

The study boasts several strengths, particularly its adherence to the robust PRISMA guidelines, ensuring a thorough and systematic approach to data collection and analysis. The comprehensive database search spanning multiple platforms like PubMed/Medline, Web of Science, and Ebsco enhances the likelihood of including all pertinent studies. The use of the Critical Appraisal Skills Programme (CASP) checklist and independent reviewers to evaluate the quality of included studies further supports the credibility of the findings.

Limitations

The significant heterogeneity in study designs and reported data could limit the comparability and synthesis of the findings. The focus on Saudi Arabia, while providing regional insights, might not be applicable in other settings, reducing the wider applicability of the results. Lastly, limiting the review to English-language articles could omit significant research published in other languages, particularly Arabic, which might be highly relevant in the regional context of Saudi Arabia.

## Conclusions

The present systematic review provided comprehensive information on the prevalence, etiology, and pattern of MFBF among children and adolescents in Saudi Arabia. It was evident that MFBF shows a male prevalence, with falls and RTAs as the main etiological factors. The mandible was the most commonly fractured bone in the maxillofacial region, often accompanied by dental involvement in children and adolescents. To decrease the occurrence of MFBF in children and adolescents, effective programs and methods to enhance parental awareness should be developed.
